# Supplementary material for: Common Variation in the PIN1 Locus Increases the Genetic Risk to Suffer from Sertoli Cell-Only Syndrome
Source: J Pers Med. 2022 Jun 4;12(6):932. doi: 10.3390/jpm12060932 (PMC9225465; doi:10.3390/jpm12060932)
Supplement: Supplementary file 1 [file jpm-12-00932-s001.zip › jpm-1713323 - file S1.docx]

**Supplementary File S1:** Authors’ group lists:

**Lisbon Clinical Group co-authors**: Carlos Calhaz-Jorge, Ana Aguiar, Sandra Sousa (Departamento de Obstetrícia, Ginecologia e Medicina da Reprodução, Hospital de Santa Maria, Centro Hospitalar Universitário de Lisboa Norte, Lisbon, Portugal; calhaz.jorge@chln.min-saude.pt, ana.aguiar.sc@gmail.com, sandrasfsousa@gmail.com), and Sónia Correia (Centro de Medicina Reprodutiva, Maternidade Alfredo da Costa, Centro Hospitalar Universitário de Lisboa Central, Lisbon, Portugal; sonia.correia2@chlc.min-saude.pt).

**IVIRMA Group co-authors**: Alberto Pacheco (IVIRMA Madrid, Spain; Alberto.Pacheco@ivirma.com), Cristina González (IVIRMA Sevilla, Spain; cristina.gonzalez@ivirma.com), Susana Álves (IVIRMA Lisbon, Portugal; Susana.Alves@ivirma.com), David Amorós (IVIRMA Barcelona, Spain; David.Amoros@ivirma.com), Jesús Aguilar (IVIRMA Vigo, Spain; Jesus.Aguilar@ivirma.com), and Fernando Quintana (IVIRMA Bilbao, Spain; Fernando.Quintana@ivirma.com).

All authors have read and agreed to the published version of the manuscript.
