# Supplementary figures and images for: Common Variation in the PIN1 Locus Increases the Genetic Risk to Suffer from Sertoli Cell-Only Syndrome
Source: J Pers Med. 2022 Jun 4;12(6):932. doi: 10.3390/jpm12060932 (PMC9225465; doi:10.3390/jpm12060932)

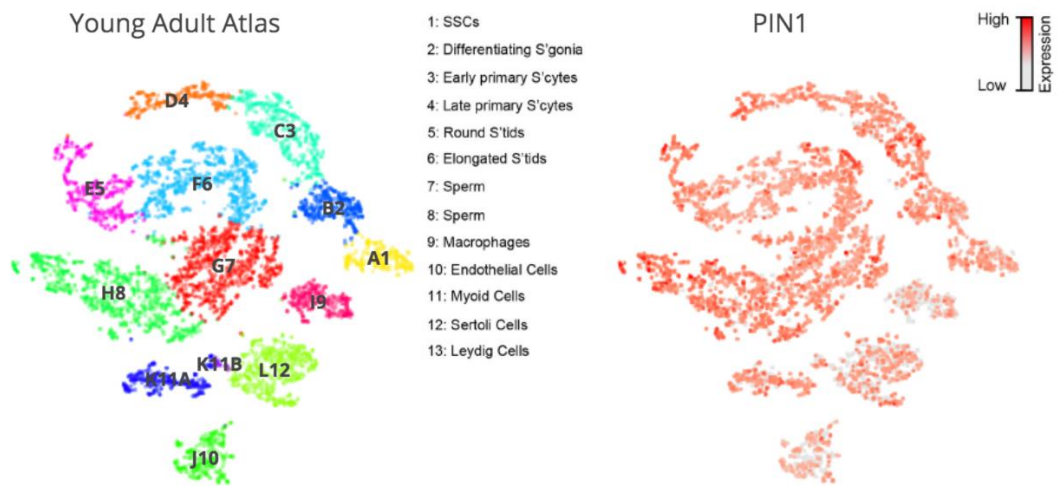

**Figure S1.** Single-cell *PIN1* expression patterns in human adult testis (extracted from Guo et al. [19]).

Supplement: Supplementary file 1 [file jpm-12-00932-s001.zip › Supplementary Figure S1.pdf]
